# Flow Dynamics of Bilateral Superior Cavopulomonary Shunts Influence Outcomes After Fontan Completion

**DOI:** 10.1007/s00246-020-02318-x

**Published:** 2020-03-10

**Authors:** Masamichi Ono, Melchior Burri, Benedikt Mayr, Lisa Anderl, Julie Cleuziou, Martina Strbad, Alfred Hager, Jürgen Hörer, Rüdiger Lange

**Affiliations:** 1 Department of Pediatric and Congenital Heart Surgery, German Heart Center Munich, Technische Universität München, Lazarettstraße 36, 80636 Munich, Germany; 2 Department of Cardiovascular Surgery, German Heart Center Munich, Technische Universität München, Lazarettstraße 36, 80636 Munich, Germany; 3 Department of Pediatric Cardiology and Congenital Heart Disease, German Heart Center Munich, Technische Universität München, Lazarettstraße 36, 80636 Munich, Germany; 4 German Center for Cardiovascular Research, Munich, Germany

## Abstract

Bilateral superior vena cava (SVC), which occurs following bilateral bidirectional cavopulmonary shunt (BCPS), is an anomaly marked by unique hemodynamics. This study aimed to determine its effects on outcomes after Fontan completion. Among 405 patients who underwent BCPS and total cavopulmonary connection (TCPC) between 1997 and 2017, 40 required a bilateral-BCPS. The dominant SVC prior to TCPC was identified according to the direction of blood flow in the central pulmonary artery, and its relationship to the inferior vena cava (IVC) was classified as a concordant or discordant relationship. Preoperative factors were analyzed to identify the risk factors for specific adverse outcomes. The length of intensive care unit (ICU) stay after TCPC was longer in the 40 patients who underwent bilateral-BCPS than in those who underwent unilateral BCPS (*p* = 0.024), and the survival rate was lower in the former group than in the latter group (*p* = 0.004). In the patients who underwent bilateral-BCPS, the dominant SVC was concordant with the IVC in 30 patients and discordant in 10 patients. With regard to whether certain morphological, hemodynamic, and flow dynamics-related variables were risk factors for adverse outcomes following TCPC, a discordant relationship between dominant SVC and IVC was identified as an independent risk factor for both a longer ICU stay (*p* = 0.037, HR 2.370) and worse survival (*p* = 0.019, HR 13.880). Therefore, in patients with a bilateral SVC who have previously undergone bilateral-BCPS, a discordant relationship between dominant SVC and IVC might contribute to worse outcomes following TCPC.

**Electronic supplementary material:**

The online version of this article (10.1007/s00246-020-02318-x) contains supplementary material, which is available to authorized users.

## Introduction

Anomalies of systemic venous drainage (ASVD) were once thought to be a contraindication for Fontan procedures [1]. However, evolving surgical and management practices have since ensured the feasibility of Fontan procedures in patients with such defects [2–5]. The development of a staged approach involving the creation of a bilateral bidirectional cavopulmonary shunt (BCPS) has significantly improved surgical outcomes for patients with a complex single ventricle, including those with ASVD [6, 7]. However, several studies have demonstrated that patients who have undergone bilateral BCPS are at greater postoperative risk for thrombus formation, unfavorable growth of the central pulmonary artery (PA), and mortality [8–10]. A bilateral SVC may pose a technical challenge with regard to the performance of a BCPS, and also causes differences in blood flow in the central PA when compared with that observed in patients who undergo a standard BCPS.

These unusual flow dynamics could influence outcomes not only after a BCPS but also after Fontan completion by means of total cavopulmonary connection (TCPC), as the flow patterns in the Fontan pathway might be different from those observed following a standard TCPC. We recently reported that ASVD carries a significant risk of a long intensive care unit (ICU) stay following TCPC, and in this setting, the most frequently observed anomaly is a bilateral SVC [11]. Thus, we hypothesize that flow dynamics in patients with a bilateral SVC might influence operative outcomes following TCPC.

Patients with a bilateral SVC are frequently found to have concomitant conditions and anatomic defects such as heterotaxy, dextrocardia, common atrioventricular valves (CAVVs), azygos continuation/interrupted inferior vena cava (IVC), and anomalous pulmonary venous drainage. These patients are considered high-risk candidates for the Fontan procedure, as each of the above factors might influence the outcomes following TCPC [3, 5, 12].

In the present study, we analyzed patients with functional single ventricles, each of whom underwent BCPS and TCPC, to compare the outcomes of the bilateral and unilateral BCPS. We subsequently focused on the patients who underwent bilateral BCPS to determine whether outcomes after TCPC were influenced by the following risk factors: patient characteristics, anatomic factors, hemodynamic variables, and pre-TCPC flow dynamics.

## Methods

### Patient Selection

The institutional review board of the Technical University of Munich approved the study. The medical records of all the patients who underwent staged single-ventricle surgical palliation at the German Heart Center Munich between 1997 and 2017 were subsequently reviewed, and 405 patients who underwent both a BCPS and TCPC were identified. Because the impact of the bilateral BCPS on post-TCPC outcomes was the focus of the study, we excluded 115 patients who did not complete these staged procedures. In-hospital and outpatient notes, echocardiographic examination results, and cardiac catheterization study reports were collected for the purposes of the investigation.

### Surgical Techniques and Perioperative Management

The surgical techniques used for the BCPS were described in a previous report [13]. The bilateral BCPS was performed only in patients with bilateral SVCs with no innominate veins and sufficiently large SVCs. The BCPS performed for persistent SVC was similar to the unilateral BCPS. In the bilateral BCPS, the right atrium and both SVCs were usually cannulated. The surgical techniques used to perform TCPC were also described in a previous report [14]. Fenestration was not routinely performed at the time of TCPC, as this procedure was performed only in high-risk patients [15].

All patients were extubated in the ICU postoperatively, and their charts were reviewed to determine postoperative ventilation times, re-intubation rates, maximum catecholamine doses, and volumes administered during the early postoperative period. Data pertaining to several postoperative parameters measured serially, including mean arterial pressure (MAP), central venous pressure (CVP), arterial partial carbon dioxide pressure (CO2), and arterial oxygen saturation (SO2), were also collected. The inotropic score was calculated using a formula described in a previous study [16]. Each patient’s volume requirement was defined as the total quantity of crystalloids and blood products infused (in ml/kg) during the first 24 h in the ICU.

### Follow-Up Surveillance

Post-discharge outcomes were determined from outpatient clinic documentation in the electronic patient charts of the German Heart Center Munich or by direct correspondence with the referring pediatric cardiologist. Mortality was ascertained at each follow-up site through direct contact with the patients’ families and their attending physicians.

### Pre-TCPC Flow Dynamics and Bilateral BCPS Pathway Dimensions

**Fig. 1:**
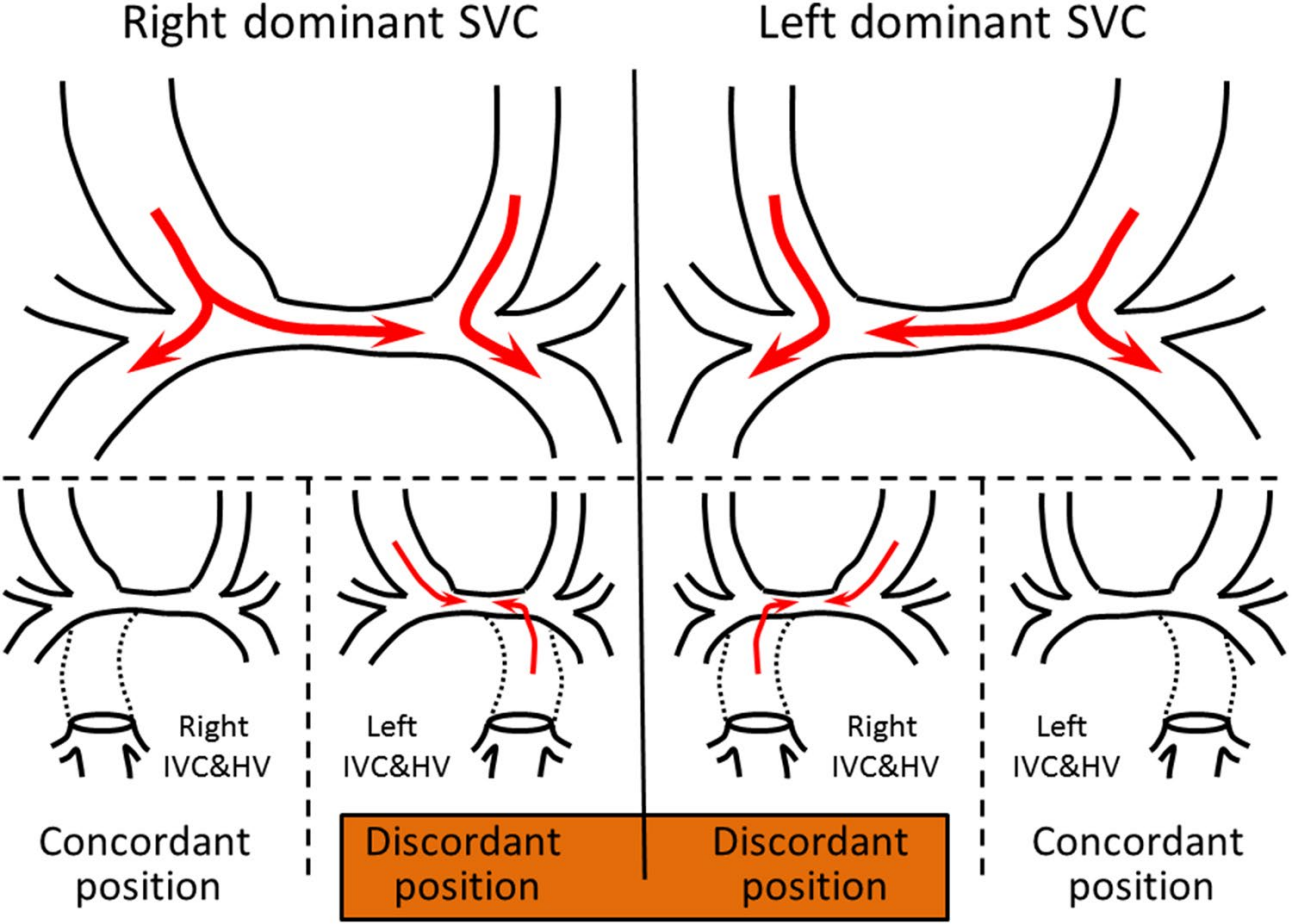
Definitions of concordant/discordant relationships between the dominant SVC and the IVC in patients with bilateral SVCs. A right-dominant SVC joins a central PA whose flow is right to left, and a left-dominant SVC joins a central PA whose flow is left to right. A concordant relationship exists when the dominant SVC and the IVC/HV are located on the same side, and a discordant relationship exists when the dominant SVC and the IVC are located on opposite sides. *SVC* superior vena cava, *IVC* inferior vena cava, *PA* pulmonary artery, *HV* hepatic vein

We evaluated the flow dynamics of the central PA in patients undergoing bilateral BCPS with pre-TCPC angiography (Fig. 1). Patients in whom the flow in the central PA was right to left (blood flow in the right SVC was directed to the right and left PA) were categorized as having a “right-dominant SVC” (Supplemental video 1), while patients in whom the flow in the central PA was left to right (blood flow in the left SVC was directed to right and left PA) were categorized as having a “left-dominant SVC” (Supplemental video 2). We also determined the location of the IVC via pre-TCPC angiography, and classified its anatomical relationship to the dominant SVC as concordant or discordant. Additionally, we measured the diameter of the right SVC, left SVC, right PA, left PA, and central PA, as well as the distance between the right SVC and left SVC, using pre-TCPC angiography. Post-TCPC outcomes were analyzed using these parameters.

### Statistical Analysis

Categorical data are presented as absolute numbers and percentages; significant differences were analyzed by the chi-square test. Continuous variables are expressed as means ± standard deviations (SDs) or as medians with interquartile ranges (IQRs), as appropriate. The independent-samples *t* test was used to compare normally distributed variables, while the Mann–Whitney *U* test was used to compare non-normally distributed variables. Kaplan–Meier curves were plotted to estimate ICU discharge probability and overall survival. Time zero was defined as the date of TCPC, and failure was defined as heart transplantation or death within the study period. Patients who were still alive and did not require a transplant were censored at the end of the study period. Risk factors were assessed with Cox regression analysis. Variables with a level of significance of less than 0.1 on univariate analysis were entered into the multiple logistic regression models, and significance was set as *p* < 0.05. All calculations were performed using standard software (SPSS version 22.0 for Windows; IBM Corp., Armonk, NY, USA).

## Results

### Patient Characteristics

A total of 405 patients underwent a BCPS (bilateral, 40; unilateral, 365) and TCPC during the study period, and the baseline characteristics are shown in Table 1. With regard to concomitant anomalies, patients in the group that underwent bilateral BCPS were more likely to have double-outlet right ventricle (DORV, *p* < 0.001), dextrocardia (*p* < 0.001), heterotaxy (*p* < 0.001), azygos continuation and interrupted IVC (*p* < 0.001), anomalous pulmonary venous drainage (*p* < 0.001), a CAVV (*p* < 0.001), a dominant right ventricle (*p* < 0.001), and extra-cardiac anomalies (*p* = 0.013) than patients who underwent unilateral BCPS.

**Table 1:** Patient population characteristics

Variables	Bilateral BCPS	Unilateral BCPS	*p* value
	*n* (%)	*n* (%)	
Number of patients	40 (9.9)	365 (90.1)	
Primary diagnosis			
HLHS	5 (12.5)	118 (32.3)	**0.010**
Univentricular heart	26 (65.0)	72 (19.7)	** < 0.001**
TA	0 (0.0)	63 (17.3)	**0.004**
DILV	2 (5.0)	48 (13.2)	0.137
PAIVS	0 (0.0)	19 (5.2)	0.139
ccTGA	0 (0.0)	17 (4.7)	0.163
Unbalanced AVSD	2 (5.0)	11 (3.0)	0.499
Other	5 (12.5)	17 (4.7)	**0.038**
Associated cardiac anomaly			
TGA	18 (45.0)	122 (33.4)	0.144
DORV	14 (35.0)	41 (11.2)	**< 0.001**
CoA	5 (12.5)	54 (14.8)	0.696
Dextrocardia	13 (32.5)	21 (5.8)	**< 0.001**
Heterotaxy	16 (40.0)	13 (3.6)	**< 0.001**
Azygos continuation	7 (17.5)	3 (0.8)	**< 0.001**
TAPVC/PAPVC	8 (20.0)	17 (4.7)	**< 0.001**
CAVV	17 (42.5)	22 (6.0)	**< 0.001**
Dominant RV	35 (87.5)	200 (54.8)	**< 0.001**
Extra-cardiac anomaly	9 (23.1)	34 (9.9)	**0.013**
Genetic anomaly	2 (5.1)	10 (2.9)	0.448

*BCPS* bidirectional cavopulmonary shunt, *HLHS* hypoplastic left heart syndrome, *TA* tricuspid atresia, *DILV* double-inlet left ventricle, *PAIVS* pulmonary atresia with intact ventricular septum, *ccTGA* congenital corrected transposition of the great arteries, *AVSD* atrioventricular septal defect, *TGA* transposition of the great arteries, *DORV* double-outlet right ventricle, *CoA* coarctation of the aorta, *TAPVC* total anomalous pulmonary vein connection, *PAPVC* partial anomalous pulmonary vein connection, *CAVV* common atrioventricular valve, *RV* right ventricle

Bold indicates *p* < 0.05

The BCPS-related data are summarized in Table 2. The data for the two groups were largely similar. The median ages at the BCPS for the bilateral and unilateral groups were 0.6 and 0.4 years, respectively (*p* = 0.156). Cardiopulmonary bypass (CPB) time was longer in patients who underwent bilateral BCPS than in those who underwent unilateral BCPS (*p* < 0.001).

**Table 2:** BCPS procedural data

Variables *n* (%), median (IQR), or mean ± SD	Bilateral BCPS	Unilateral BCPS	*p* value
Number of patients	40 (9.9)	365 (90.1)	
Age at BCPS (years)	0.6 (0.4–1.5)	0.4 (0.3–0.8)	0.156
Weight at BCPS (kg)	6.5 (5.0–8.2)	5.8 (4.9–7.4)	0.337
Height at BCPS (cm)	69 (61–78)	64 (60–70)	0.056
Pre-BCPS catheterization data			
Hemoglobin level	14.7 ± 2.2	14.2 ± 2.0	0.186
Pulmonary artery pressure	14.7 ± 4.6	14.8 ± 4.8	0.913
Left atrial pressure	6.2 ± 2.7	6.2 ± 2.7	0.949
Transpulmonary gradient (TPG)	8.3 ± 3.7	8.5 ± 4.3	0.863
Systemic ventricular pressure	78.1 ± 11.4	79.9 ± 12.6	0.418
End-diastolic pressure (EDP)	7.9 ± 2.9	8.6 ± 3.1	0.210
Systolic aortic pressure	76.6 ± 11.0	78.0 ± 12.7	0.534
Mean aortic pressure (MAP)	53.2 ± 8.1	50.9 ± 8.4	0.129
Aortic oxygen saturation (SO2)	78.3 ± 7.1	75.3 ± 7.7	**0.032**
Operative data			
CPB time (min)	92 (68–113)	61 (45–85)	**< 0.001**
Aortic cross-clamp	5 (12.5)	77 (22.1)	0.158
Concomitant procedure			
DKS	1 (2.5)	3 (0.8)	0.308
AVV procedure	1 (2.5)	41 (11.2)	0.085
Aorta patch plasty	1 (2.5)	10 (2.7)	0.929
PA reconstruction	13 (32.5)	143 (39.2)	0.410
SVOT enlargement	0 (0.0)	6 (1.6)	0.414

*BCPS* bidirectional cavopulmonary shunt, *IQR* interquartile ranges, *SD* standard deviations, *CPB* cardiopulmonary bypass, *DKS* Damus–Kaye–Stansel anastomosis, *AVV* atrioventricular valve, *PA* pulmonary artery, *SVOT* systemic ventricular outflow tract

Bold indicates *p* < 0.05

The TCPC-related data for each group are summarized in Supplementary Table S1. The median ages at TCPC for the bilateral and unilateral groups were 2.6 and 2.0 years, respectively (*p* = 0.089). Hemoglobin levels (16.8 vs. 16.0 g/dl; *p* = 0.010), mean PA pressure (10.0 vs. 9.1 mmHg; *p* = 0.017), and mean LAP (6.6 vs. 5.3 mmHg; *p* = 0.001), were higher in patients who underwent bilateral BCPS than in patients who underwent unilateral BCPS. Additionally, CPB time (*p* = 0.003) was longer, and aortic cross-clamping (*p* = 0.003) was more frequently needed in the bilateral BCPS group than in the unilateral BCPS group.

### Post-TCPC Outcomes

Five patients died in the ICU within 30 days after TCPC (30-day mortality: bilateral BCPS, 2.5% [1/40]; unilateral BCPS, 1.1% [4/395]; *p* = 0.445). Kaplan–Meier curves comparing times to discharge from the ICU after TCPC are plotted in Supplementary Figure S1. Patients who underwent bilateral BCPS spent more time in the ICU than patients who underwent unilateral BCPS (*p* = 0.024, log-rank test).

Of the 400 patients who survived the early postoperative period, six (1.5%) were lost to follow-up after hospital discharge. The median follow-up period for the remaining 394 patients was 6.2 years (range, 1.5–11.6 years). Eleven patients experienced late death (bilateral BCPS, 4; unilateral BCPS, 7). According to the corresponding Kaplan–Meier curves, overall survival at 15 years was lower in the bilateral BCPS group (83.9%) than in the unilateral BCPS group (96.1%; *p* = 0.004) (Supplementary Figure S2).

Cardiac reoperation after TCPC was required in 30 patients (bilateral BCPS, 2; unilateral BCPS, 28). Estimated freedom from cardiac reoperation at 10 years did not differ between the groups (bilateral BCPS, 93.2%; unilateral BCPS, 86.3%; *p* = 0.542). Catheter intervention was required in 74 patients (bilateral BCPS, 7; unilateral BCPS, 67). Freedom from catheter intervention at 10 years did not differ between the groups (bilateral BCPS, 73.2%; unilateral BCPS, 76.3%; *p* = 0.893).

### Flow Dynamics and Bilateral BCPS Pathway Dimensions

Data regarding flow dynamics and BCPS pathway dimensions in the 40 patients who underwent bilateral BCPS are shown in Table 3. According to the definition provided in the Methods section, a concordant relationship between the dominant SVC and the IVC was observed in 30 patients (both were in the right position in 21 patients, and both were in the left position in 9 patients), and a discordant relationship was observed in 10 patients (a left-dominant SVC and right IVC were observed in 9 patients, and a right-dominant SVC and left IVC were observed in 1 patient).

**Table 3:** Comparison of SVC/IVC configurations in patients who underwent bilateral BCPS

Variables Mean ± SD	Concordant relationship	Discordant relationship	*p *value
Number of patients	30	10	

	Right	Left	Right	Left	
Dominant SVC	21	9	1	9	
IVC/HV to PA connection	21	9	9	1	
Central PA diameter	7.2 ± 3.5		6.3 ± 2.1		0.431
Right PA diameter	10.3 ± 2.4		9.7 ± 2.7		0.532
Left PA diameter	9.5 ± 2.0		10.0 ± 3.7		0.728
Distance from right/left SVCs	17.5 ± 5.7		23.4 ± 12.8		0.057
Dominant SVC diameter	8.9 ± 1.7		10.8 ± 3.2		0.090
Non-dominant SVC diameter	8.8 ± 2.3		7.8 ± 1.3		0.192
Dominant/non-dominant SVC ratio	1.1 ± 0.3		1.4 ± 0.3		**0.012**

Diameters expressed in millimeters (mm)

*SVC* superior vena cava, *IVC* inferior vena cava, *BCPS* bidirectional cavopulmonary shunt, *SD* standard deviations, *HV* hepatic vein, *PA* pulmonary artery

Bold indicates *p* < 0.05

The diameters of the central PA (*p* = 0.431), right PA (*p* = 0.532), left PA (*p* = 0.728), dominant SVC (*p* = 0.090), and non-dominant SVC (*p* = 0.192) did not differ between the groups (Table 5), nor did the distance between the right SVC and left SVC (*p* = 0.057). However, the dominant SVC-to-non-dominant SVC ratio was higher (*p* = 0.012) in patients with discordant anatomy than in patients with concordant anatomy.

### Impact of a Discordant Relationship Between the Dominant SVC and the IVC on Post-TCPC Outcomes

**Fig. 2:**
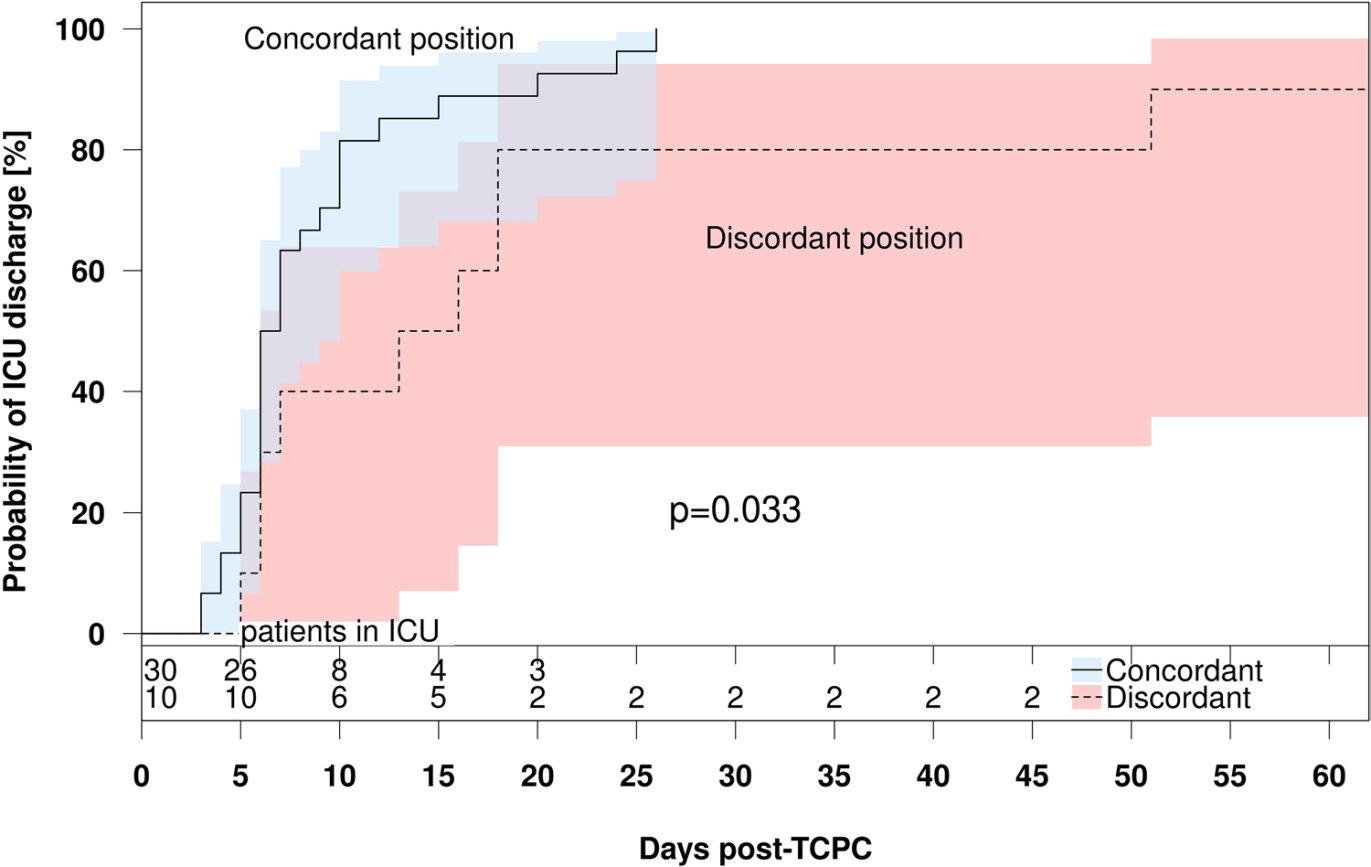
Kaplan–Meier curves comparing time to discharge from the ICU after TCPC between patients with concordant and discordant SVC/IVC configurations. *Note* time to ICU discharge was significantly longer in patients with discordant anatomy than in those with concordant anatomy (*p* = 0.033, log-rank test). *ICU* intensive care unit, *TCPC* total cavopulmonary connection, *SVC* superior vena cava, *IVC* inferior vena cava

We determined whether morphological factors, hemodynamic factors, pre-TCPC variables, BCPS pathway size, and flow dynamics-related variables were risk factors for a prolonged ICU stay in the 40 patients who underwent bilateral BCPS (Table 4). Multivariate analysis showed that a discordant relationship between the dominant SVC and the IVC was an independent risk factor for a longer stay (*p* = 0.037, HR 2.370). The average ICU stay was longer (*p* = 0.033) in patients with discordant anatomy (*n* = 10) than in those with concordant anatomy (*n* = 30) (Fig. 2).

**Table 4:** Influence of morphological hemodynamic and flow dynamics-related factors on length of ICU stay after TCPC

Variables	Univariate model			Multivariate model		
	*p* value	HR	95% CI	*p* value	HR	95% CI
Morphological factors						
DORV	0.278	0.692	0.355–1.348			
Dextrocardia	0.579	0.822	0.413–1.639			
Heterotaxy	0.485	1.263	0.656–2.427			
Azygos continuation	0.175	1.842	0.763–4.444			
TAPVD/PAPVD	0.269	1.597	0.696–3.676			
CAVV	0.671	1.147	0.609–2.160			
Dominant RV	0.401	1.513	0.576–3.984			
Hemodynamic factors						
PAP	0.580	10.28	0.933–1.133			
LAP	0.926	1.005	0.897–1.126			
SVP	0.899	1.002	0.975–1.030			
EDP	0.920	1.006	0.890–1.138			
AVVR pre-TCPC	0.061	2.331	0.961–5.650	0.050	1.044	1.001–6.024
APC	0.217	1.587	0.763–3.300			
Pre-TCPC data						
Age at TCPC	0.097	1.106	0.982–1.245			
CPB time	0.113	1.006	0.999–1.013			
Aortic cross-clamp	0.137	1.642	0.854–3.155			
Aortic cross-clamp time	0.073	1.019	0.998–1.041			
BCPS pathway size						
Small central PA	0.198	1.592	0.784–3.226			
Diameter of right SVC	0.900	1.011	0.853–1.199			
Diameter of left SVC	0.220	1.075	0.958–1.206			
Diameter of right PA	0.757	0987	0.850–1.125			
Diameter of left PA	0.593	1.090	0.924–1.148			
Diameter of central PA	0.219	0.936	0.843–1.040			
Distance between SVCs	0.077	1.044	0.995–1.094			
TCPC conduit diameter	0.450	0.874	0.616–1.241			
BCPS flow dynamics						
Right dominant SVC (right to left)	0.405	0.758	0.395–1.456			
Left dominant SVC (left to right)	0.405	1.319	0.687–2.533			
Discordant relationship between dominant SVC and IVC	**0.043**	2.288	1.028–5.102	**0.037**	2.370	1.052–5.348

*ICU* intensive care unit, *TCPC* total cavopulmonary connection, *HR* hazard ratio, *CI* confidence interval, *DORV* double-outlet right ventricle, *TAPVC* total anomalous pulmonary venous connection, *PAPVC* partial anomalous pulmonary venous connection, *CAVV* common atrioventricular valve, *RV* right ventricle, *PAP* pulmonary arterial pressure, *LAP* left atrial pressure, *SVP* systemic ventricular pressure, *EDP* end diastolic pressure, *AVVR* atrioventricular valve regurgitation, *APC* aortopulmonary collaterals, *CPB* cardiopulmonary bypass, *BCPS* bidirectional cavopulmonary shunt, *PA* pulmonary artery, *SVC* superior vena cava

Bold indicates *p* < 0.05

We determined whether morphological factors, hemodynamic factors, flow dynamics-related variables, pre-TCPC variables, and BCPS pathway dimensions were risk factors for survival after TCPC (Table 5). Multivariate analysis demonstrated that a discordant relationship between the dominant SVC and the IVC was an independent risk factor for survival (*p* = 0.019, HR 13.880). Kaplan–Meier analysis showed that survival after TCPC was significantly lower (*p* = 0.002) in the subset of patients with discordant anatomy than in that with concordant anatomy (Supplementary Figure S3).

**Table 5:** Influence of morphological, hemodynamic, and flow dynamics-related factors on mortality after TCPC

Variables	Univariate model			Multivariate model		
	*p* value	HR	95% CI	*p* value	HR	95% CI
Morphological factors						
DORV	0.485	0.457	0.051–4.103			
Dextrocardia	0.560	0.520	0.058–4.672			
Heterotaxy	0.966	0.962	0.160–5.777			
Azygos continuation	0.268	2.751	0.459–16.487			
TAPVD/PAPVD	0.220	3.091	0.509–18.794			
CAVV	0.577	0.601	0.100–3.600			
Dominant RV	0.426	0.407	0.045–3.716			
Hemodynamic factors						
PAP	0.052	1.201	0.998–1.445			
LAP	0.629	1.063	0.830–1.362			
SVP	0.614	1.017	0.952–1.087			
EDP	0.873	1.026	0.749–1.406			
AVVR pre-TCPC	0.887	1.172	0.131–10.508			
APC	0.910	0.881	0.098–7.900			
Pre-TCPC data						
Age at TCPC	**0.013**	1.206	1.041–1.397			
CPB time	0.163	1.014	0.994–1.033			
Aortic cross-clamp	0.373	2.257	0.377–13.517			
Aortic cross-clamp time	0.590	1.009	0.976–1.043			
BCPS pathway size						
Small central PA	0.547	1.741	0.287–10.565			
Diameter of right SVC	0.605	0.882	0.548–1.420			
Diameter of left SVC	0.071	1.306	0.978–1.745			
Diameter of right PA	0.750	1.052	0.770–1-438			
Diameter of left PA	0.109	1.280	0.946–1.730			
Diameter of central PA	0.692	1.050	0.824–1.339			
Distance between SVCs	**0.017**	1.075	1.013–1.140			
TCPC conduit diameter	0.866	0.941	0.466–1.903			
BCPS flow dynamics						
CPA flow right to left	0.124	0.178	0.020–1.604			
CPA flow left to right	0.124	5.618	0.623–50.00			
Discordant relationship between dominant SVC and IVC	**0.019**	13.880	1.529–125.99	**0.019**	13.880	1.529–125.98

*TCPC* total cavopulmonary connection, *HR* hazard ratio, *CI* confidence interval, *DORV* double-outlet right ventricle, *TAPVC* total anomalous pulmonary venous connection, *PAPVC* partial anomalous pulmonary venous connection, *CAVV* common atrioventricular valve, *RV* right ventricle, *PAP* pulmonary arterial pressure, *LAP* left atrial pressure, *SVP* systemic ventricular pressure, *EDP* end diastolic pressure, *AVVR* atrioventricular valve regurgitation, *APC* aortopulmonary collaterals, *CPB* cardiopulmonary bypass, *BCPS* bidirectional cavopulmonary shunt, *PA* pulmonary artery, *SVC* superior vena cava

### Comparison of Post-TCPC Outcomes Between Patients with Concordant and Discordant Anatomy

To confirm whether a discordant relationship between the dominant SVC and the IVC is the sole intrinsic factor responsible for the poorer outcomes observed in patients with discordant anatomy, we compared pre- and post-TCPC variables between the 10 patients with discordant anatomy and the 30 patients with concordant anatomy (Supplementary Table S2). Primary diagnoses and morphological variables did not differ between the groups. Among cardiac catheterization variables, pre-TCPC SO2 was lower in patients with discordant anatomy than in patients with concordant anatomy (*p* = 0.007). Among postoperative outcomes, the incidence of secondary fenestration (*p* = 0.012) and ventricular dysfunction (*p* = 0.012) were higher among patients with discordant anatomy than among those with concordant anatomy.

We measured postoperative variables including the following hemodynamic variables at the time of ICU admission; 1 and 2 h before extubation; and 1, 2, 6, 12, and 24 h after extubation: MAP, volume administration, CO_2_, CVP, inotrope score, and SO_2_ (Supplementary Table S3). MAP was elevated after extubation in the concordant group (*p* = 0.003) but not in the discordant group (Supplementary Figure S4A). MAP at 6 h after extubation was lower in the discordant group than in the concordant group (*p* = 0.040). Volume administration decreased after extubation in the concordant group (*p* < 0.001) but not in the discordant group (Supplementary Figure S4B). Volume administration at 24 h after extubation was significantly higher in the discordant group (*p* = 0.042). CO2 was higher in the discordant group than in the concordant group, and was significant at 1 and 2 h before extubation (Supplementary Figure S4C). CVP was also higher in the discordant group and was significant at 2 h before extubation (Supplementary Figure S4D).

## Discussion

This study revealed that patients who underwent bilateral BCPS experienced longer ICU stays and poorer survival following TCPC than patients who underwent unilateral BCPS. Furthermore, subgroup analysis of the 40 patients who underwent bilateral BCPS demonstrated that a discordant relationship between the dominant SVC and the IVC was an independent risk factor for worse outcomes after TCPC. Patients with discordant anatomy demonstrated hemodynamic instability during their ICU stays following TCPC.

### Patient Morphological and Flow Dynamics Features After a Bilateral BCPS Procedure

A bilateral BCPS is generally required in patients with a bilateral SVC, although this procedure may pose its own anatomical and physiological challenges. Anastomosis of relatively small SVCs during early infancy is technically problematic and may heighten the risk of stenosis and thrombus formation [8]. During the bilateral BCPS, blood flow from each SVC is preferentially directed to the vessels of the ipsilateral lung, which makes the direction of blood flow in the central PA unpredictable. Flow stagnation at the central PA confluence from both SVCs, poor growth of the central PA, and unfavorable flow dynamics may occur prior to TCPC. During the data collection phase of this study, we noticed that blood flow in the central PA traveled in a certain direction, and that some patients with a left-to-right central PA flow and a right-sided IVC had a complicated postoperative course. Thus, we hypothesized that flow stagnation at the point where the central PA is joined by the SVC and IVC in the newly created TCPC pathway might be a risk factor for a poor outcome. Our results supported this hypothesis. We determined that a discordant relationship between the dominant SVC and the IVC was an independent risk factor for both a prolonged ICU stay and worse survival following TCPC. During the postoperative ICU stay, hemodynamic instability was also noted in patients with a discordant relationship between the dominant SVC and the IVC. In a previous study, we demonstrated that postoperative hemodynamics improved soon after extubation following TCPC [16]. However, prolonged hemodynamic instability was observed even after extubation in patients with a discordant relationship between the dominant SVC and the IVC. Furthermore, two of 10 discordant patients needed secondary fenestration, whereas none of 30 concordant patients needed secondary fenestration. These results suggest that a discordant relationship between the dominant SVC and the IVC is an intrinsic factor increasing the risk of flow stagnation in the central PA, lengthened ICU stays, and increased mortality and morbidity following TCPC.

### Other Possible Morphological Features Associated with Patient Outcomes After a Bilateral BCPS Procedure

A bilateral SVC may be a surrogate for a complex type of single ventricle. Patients who have undergone bilateral BCPS are significantly more likely to suffer from dextrocardia, heterotaxy, CAVVs, and anomalous pulmonary venous drainage than those who have undergone unilateral BCPS. Anatomical anomalies of this magnitude are very likely to worsen pre-Fontan conditions and undermine outcomes after TCPC. Although Azakie et al. showed improved Fontan procedure outcomes in patients with heterotaxy, the authors indicated that these patients still face risks [12]. Poh et al. reported poor surgical outcomes with respect to single-ventricle palliation in patients with dextrocardia [17]. The unique hemodynamics imposed by a bilateral BCPS may offer yet another explanation for such findings, as our results indicate that abnormalities pertaining to certain flow dynamics-related and laboratory parameters, such as higher preoperative PA pressures, elevated LAP, and increased hemoglobin levels, can occur at the time of TCPC in patients who have undergone bilateral BCPS. However, our results also demonstrated that none of these anatomical or hemodynamic factors was an independent risk factor for worse outcomes following TCPC in patients who had undergone bilateral BCPS.

### Controversy with Respect to the Results After a Bilateral BCPS Procedure

Nevertheless, there are reports that dispute the risks associated with the performance of bilateral BCPS. Whitehead et al. amassed magnetic resonance data showing no differences in pulmonary flow splits between patients with a bilateral SVC and those with a unilateral SVC [18]. Kogan et al. showed that the presence of a left SVC did not affect mortality and morbidity after a BCPS [19], and Ando et al. found that there was no risk of thrombosis or impaired central PA growth following bilateral BCPS; they also found that the rates of Fontan completion were comparable between patients who underwent bilateral BCPS and those who underwent unilateral BCPS [20]. Finally, Alsoufi et al. did not identify bilateral SVC as a risk factor for mortality after BCPS [21].

### Therapeutic Options and New Techniques

Various surgical modifications have been devised to improve flow dynamics after bilateral BCPS. Amodeo et al. confirmed the utility of a unifocal bilateral BCPS using a computational fluid dynamics (CFD) model [22]. This technique enabled localized admixing of blood at the confluence of the caval veins as they lead into PA, without substantial zonal recirculation. Honjo et al. described a V-shaped bilateral BCPS, which enables the creation of a larger central PA and is associated with a lower risk of re-intervention [6]. However, a large aorta may limit the ability to create a unifocal and V-shaped bilateral BCPS because it may increase the angles of the two SVCs and add tension to the anastomosis.

Two CFD studies involving patients with bilateral SVCs who underwent TCPC were performed to identify optimal pathway configurations. De Zélicourt et al. demonstrated that a large offset between the IVC and the left SVC resulted in flow stasis and unbalanced hepatic flow distributions in the conventional TCPC model and suggested that shifting the IVC and positioning it between the two SVCs resulted in a 7% decrease in power loss and eliminated the associated flow abnormalities in the central PA [23]. The authors also recommended suturing the right and left SVC closer together during the BCPS. In contrast, the CFD study by Sun et al. demonstrated that tightly anastomosing the left SVC and right SVC and connecting the extra-cardiac conduit from the IVC to the right PA would result in greater power loss and lower energy efficiency; they also showed that anastomosing the conduit from the IVC to the right PA while leaving a large distance between the left SVC and right SVC resulted in the least amount of power loss and greater energy efficiency [24]. Their results were quite different from those of de Zelicout et al., and they speculated that the conduit used in the study by de Zélicourt et al. had no obvious curve and was situated just under the right SVC, which led to interactions between the stream from the right SVC and that from the extra-cardiac conduit, resulting in chaos near the root of the right SVC. Because of the large curvature of the conduit, no obvious chaos was noted near the root of the left SVC or the right SVC when the conduit was connected to the latter vessel. The appropriate distance between the sites of anastomosis of the right SVC and left SVC has also remained a controversial subject.

With regard to new technical options, Marsden et al. tested a Y-shaped extra-cardiac Fontan baffle using a CFD model [25], and demonstrated the technical feasibility and intermediate outcomes of this modification in six patients [26]. Their results indicate that the procedure may be an option for high-risk patients (with discordant anatomy) during bilateral BCPS.

Taking all these findings into consideration, our current approach for patients with bilateral BCPS at the time of Fontan completion is as follows: (1) We check the flow direction of the central PA and determine the dominant SVC. (2) If the relation of the dominant SVC and the IVC is concordant, we perform the extra-cardiac TCPC as usual. (3) If the relation of the dominant SVC and the IVC is discordant, we reconsider the indication of Fontan completion, re-evaluating the preoperative risks such as PAP, PA size, systemic ventricular function, and AVV regurgitation. (4) Eventually, isolated procedures such as central PA reconstruction or AVV repair are also considered prior to Fontan completion. (5) Initial fenestration concomitant with Fontan completion is indicated. (6) At Fontan completion, placement of distal conduit anastomosis near the dominant SVC can be applied. (7) Finally, Y-shaped extra-cardiac Fontan conduit may be an option.

### Study Limitations

This study was limited by its retrospective and single-center design. Data availability was also a limitation, which may be attributed to differences among the follow-up periods for each patient. Advances in surgical and medical management over time also may have skewed long-term outcomes. Additionally, the small number of events might limit the reliability of the Cox regression results in some cases.

## Conclusions

Although the timing of the BCPS and that of TCPC were similar, post-TCPC ICU stays were longer, and survival following TCPC was poorer in patients who underwent bilateral BCPS than in those who underwent unilateral BCPS. The unique flow dynamics imposed by a bilateral BCPS and the presence of concomitant morphological anomalies likely influenced the hemodynamics of the Fontan procedures, conferring worse outcomes after TCPC. Subgroup analysis of the patients who underwent bilateral BCPS showed that a discordant relationship between the dominant SVC and the IVC was an independent risk factor for hemodynamic instability, prolonged ICU stays, and worse survival following TCPC, and might be an intrinsic risk factor for such outcomes.

## Electronic supplementary material

Below is the link to the electronic supplementary material.

Supplementary file1 (TIF 1319 kb)

Supplementary file2 (TIF 1236 kb)

Supplementary file3 (TIF 1194 kb)

Supplementary file4 (JPG 55 kb)

Supplementary file5 (JPG 44 kb)

Supplementary file6 (JPG 46 kb)

Supplementary file7 (JPG 47 kb)

Supplementary file8 (DOCX 42 kb)

Supplementary file9 (MP4 345 kb)

Supplementary file10 (MP4 382 kb)

Supplementary file11 (MP4 311 kb)

Supplementary file12 (MP4 198 kb)

Supplementary file13 (DOCX 42 kb)

## Abbreviations

ASVD: Anomalies of the systemic venous drainage
APC: Aortopulmonary collaterals
BCPS: Bidirectional cavopulmonary shunt
CAVV: Common atrioventricular valve
CFD: Computational fluid dynamics
CO2: Partial carbon dioxide pressure
CRP: C-reactive protein
CVP: Central venous pressure
DORV: Double-outlet right ventricle
ICU: Intensive care unit
IVC: Inferior vena cava
IQR: Interquartile range
MAP: Mean arterial pressure
PA: Pulmonary artery
PAVM: Pulmonary arteriovenous malformation
PLE: Protein losing enteropathy
SD: Standard deviation
SO2: Arterial oxygen saturation
SVC: Superior vena cava
TCPC: Total cavopulmonary connection
