# Supplementary figures and images for: Flow Dynamics of Bilateral Superior Cavopulomonary Shunts Influence Outcomes After Fontan Completion
Source: Pediatr Cardiol. 2020 Mar 10;41(4):816–26. doi: 10.1007/s00246-020-02318-x (PMC7256021; doi:10.1007/s00246-020-02318-x)

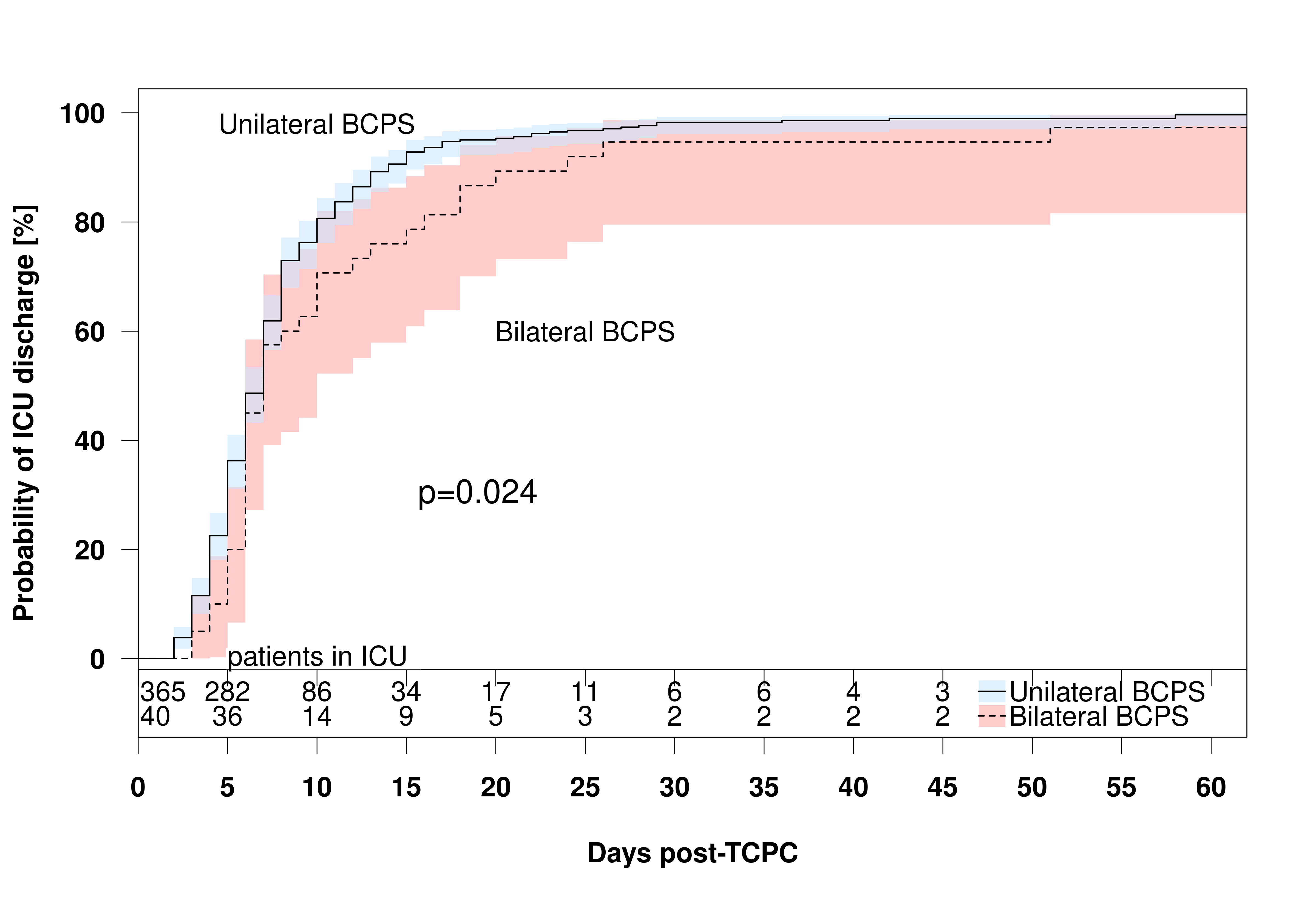

Supplement: Supplementary file 1 — Supplementary file1 (TIF 1319 kb) [file 246_2020_2318_MOESM1_ESM.tif]

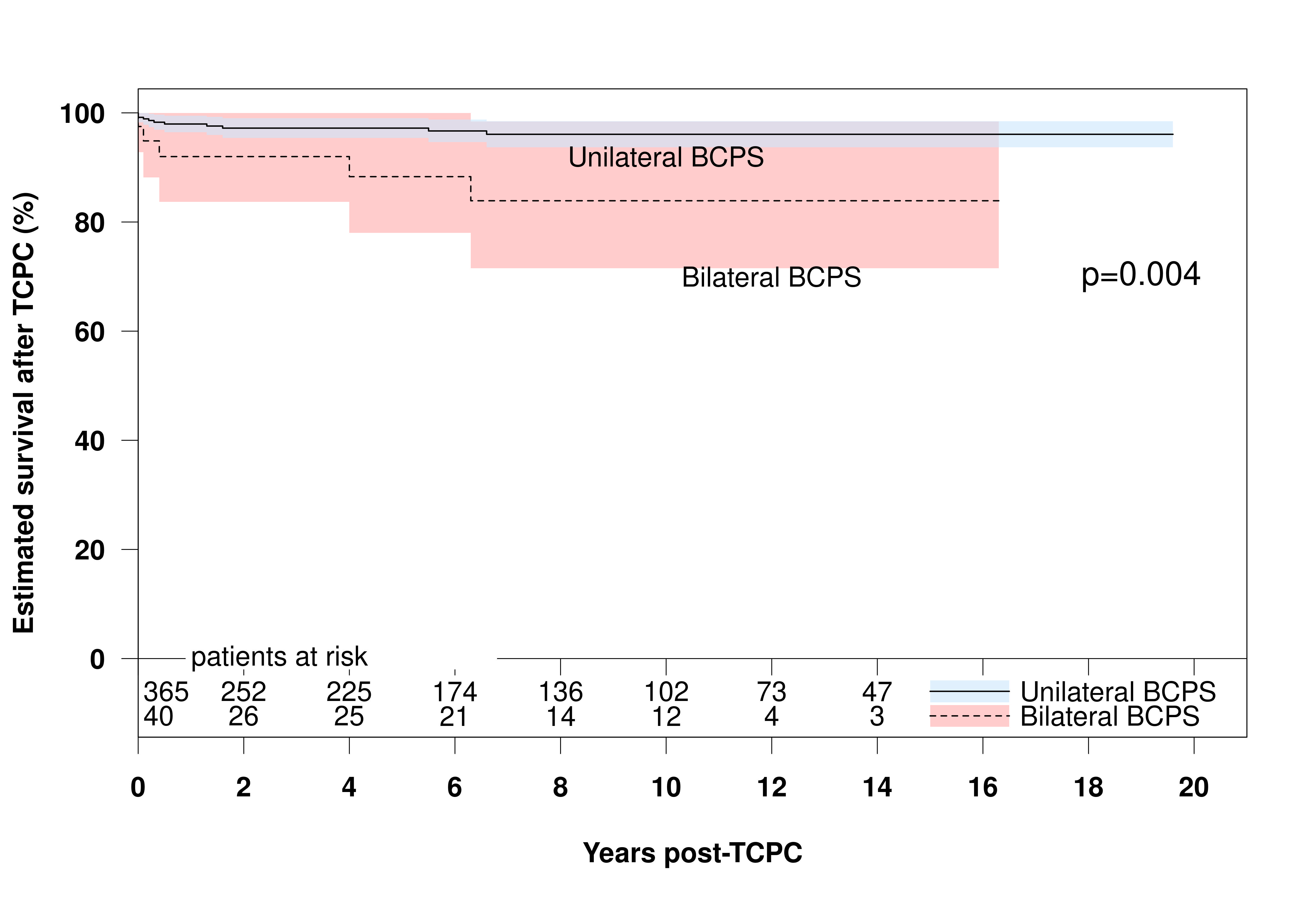

Supplement: Supplementary file 2 — Supplementary file2 (TIF 1236 kb) [file 246_2020_2318_MOESM2_ESM.tif]

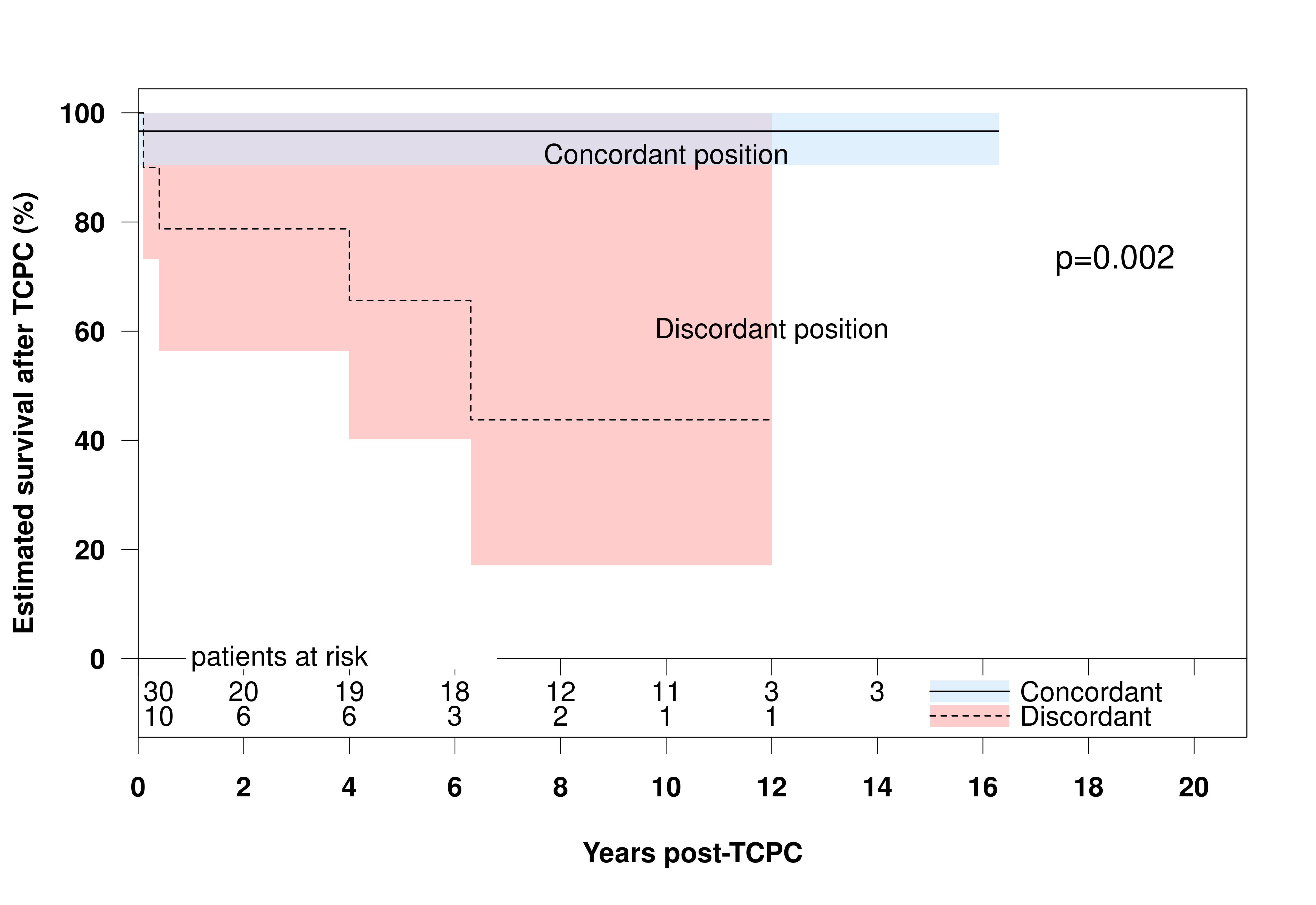

Supplement: Supplementary file 3 — Supplementary file3 (TIF 1194 kb) [file 246_2020_2318_MOESM3_ESM.tif]

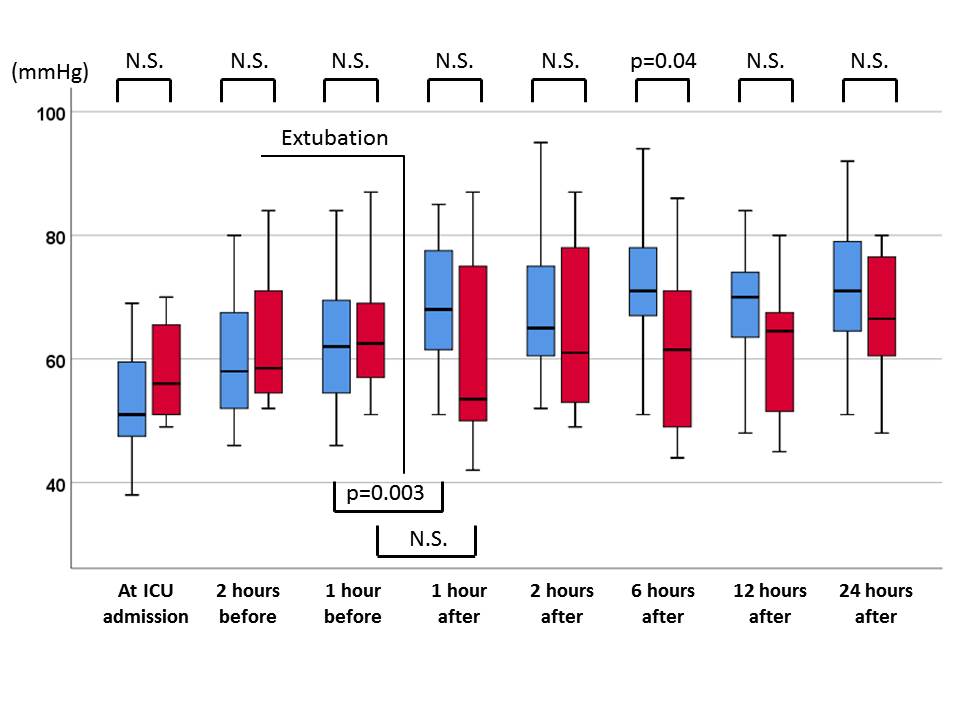

Supplement: Supplementary file 4 — Supplementary file4 (JPG 55 kb) [file 246_2020_2318_MOESM4_ESM.jpg]

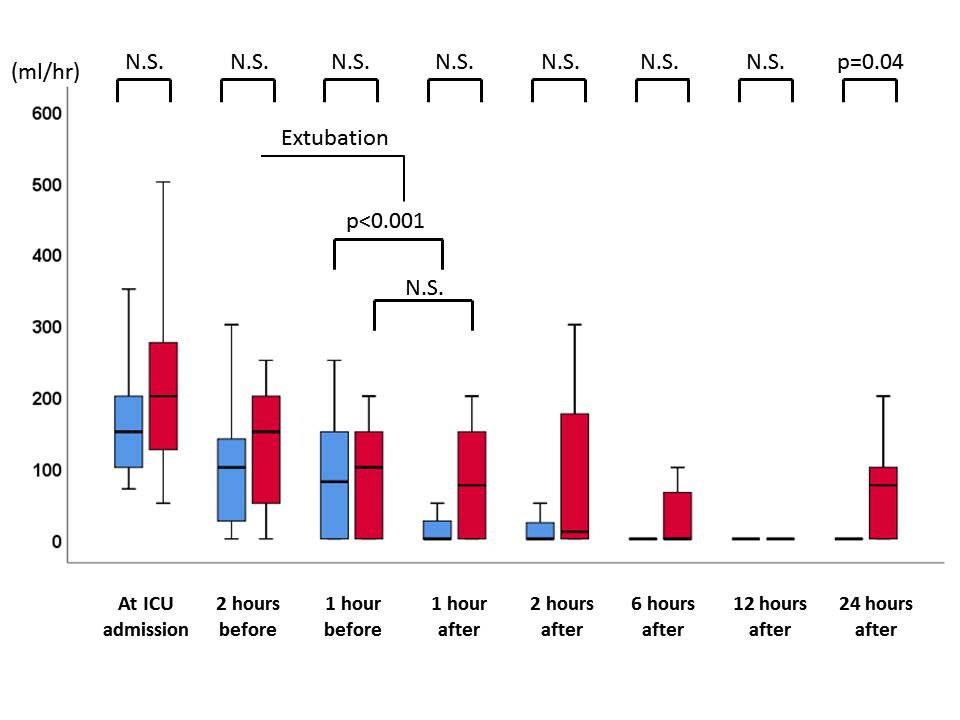

Supplement: Supplementary file 5 — Supplementary file5 (JPG 44 kb) [file 246_2020_2318_MOESM5_ESM.jpg]

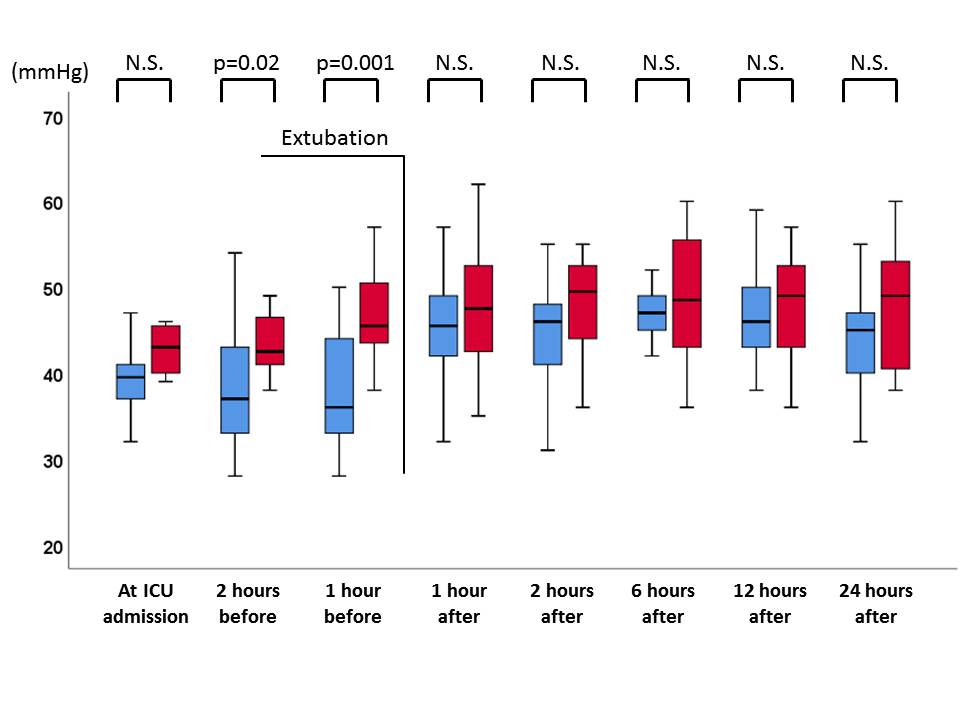

Supplement: Supplementary file 6 — Supplementary file6 (JPG 46 kb) [file 246_2020_2318_MOESM6_ESM.jpg]

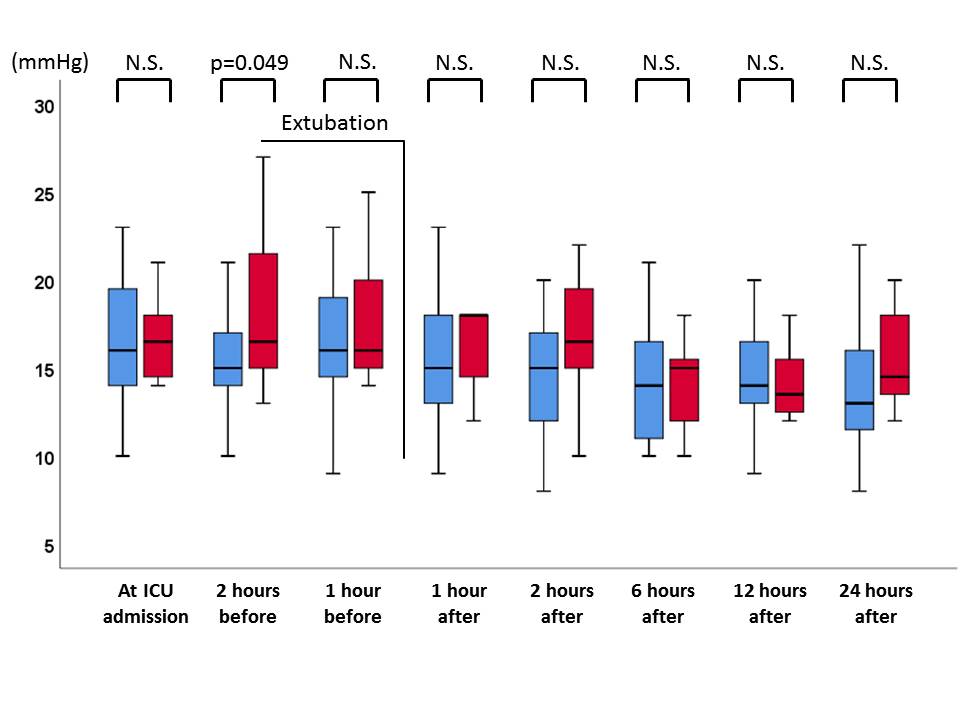

Supplement: Supplementary file 7 — Supplementary file7 (JPG 47 kb) [file 246_2020_2318_MOESM7_ESM.jpg]
