# Supplementary material for: Flow Dynamics of Bilateral Superior Cavopulomonary Shunts Influence Outcomes After Fontan Completion
Source: Pediatr Cardiol. 2020 Mar 10;41(4):816–26. doi: 10.1007/s00246-020-02318-x (PMC7256021; doi:10.1007/s00246-020-02318-x)
Supplement: Supplementary file 8 — Supplementary file8 (DOCX 42 kb) [file 246_2020_2318_MOESM8_ESM.docx]

**Supplementary File**

**Tables**

| **Table S1**: TCPC procedural data | |  |  |  |
| --- | --- | --- | --- | --- |
| Variables | | Bilateral BCPS | Unilateral BCPS | *P*-value |
| n (%), median (IQR), or mean ± SD | |  |  |  |
| Number of patients | | 40 (9.9) | 365 (90.1) |  |
| Age at TCPC (year) | | 2.6 (2.2-3.1) | 2.0 (1.7-2.7) | 0.089 |
| Interval between BCPC and TCPC | | 1.8 (1.2-2.4) | 1.5 (1.1-2.0) | 0.160 |
| Weight at TCPC (kg) | | 11.5 (10.6-12.8) | 11.4 (10.5-12.8) | 0.476 |
| Height at TCPC (cm) | | 89 (85-94) | 86 (82-91) | 0.134 |
| Pre-TCPC catheterization data | |  |  |  |
|  | Hemoglobin level | 16.8±2.5 | 16.0±1.7 | **0.010** |
|  | Pulmonary artery pressure | 10.0±3.6 | 9.1±2.7 | **0.017** |
|  | Left atrial pressure | 6.6 ± 3.1 | 5.3 ± 2.3 | **0.001** |
|  | Transpulmonary gradient (TPG) | 3.3±2.0 | 3.7±1.7 | 0.191 |
|  | Systemic ventriclar pressure | 82.1 ± 12.4 | 81.7 ± 13.3 | 0.873 |
|  | End-diastolic pressure (EDP) | 8.6 ± 2.6 | 7.2 ± 2.7 | 0.099 |
|  | Systolic Aortic pressure | 80.9 ± 12.8 | 78.6 ± 14.0 | 0.343 |
|  | Mean Aortic pressure (MAP) | 58.7 ± 10.5 | 55.7 ± 10.8 | 0.123 |
|  | Aortic oxygen saturation (SO2) | 84.3±6.6 | 82.1±7.0 | 0.080 |
| Operative data | |  |  |  |
|  | Intracardiac TCPC | 1 (2.5) | 21 (5.8) | 0.389 |
|  | Extracardiac TCPC | 39 (97.5) | 344 (94.2) |  |
|  | CPB time (min) | 83 (55-133) | 59 (44-82) | **0.003** |
|  | Aortic cross-clamp | 17 (42.5) | 79 (21.6) | **0.003** |
|  | Aortic cross-clamp time (min) | 34 (15-59) | 41 (25-68) | 0.388 |
|  | Concomitant procedure | 17 (42.5) | 91 (24.9) | **0.017** |
|  | DKS | 0 (0.0) | 4 (1.1) | 0.506 |
|  | AVV procesure | 10 (25.0) | 50 (13.7) | 0.056 |
|  | PA reconstruction | 6 (15.0) | 33 (9.0) | 0.225 |
|  | Atrioesptectomy | 3 (7.5) | 5 (1.4) | **0.008** |
|  | Fenestration | 1 (2.5) | 12 (3.3) | 0.788 |

Bold indicates P<0.05

TCPC: total cavopulmonary connection; BCPS: bidirectional cavopulmonary shunt;

IQR: interquartile ranges; SD: standard deviations;

CPB: cardiopulmonary bypass; DKS: Damus-Kaye-Stansel anastomosis; AVV: atrioventricular valve

PA: pulmonary artery;

| **Table S2**: Comparison of post-TCPC outcomes between patients with concordant and discordant anatomy | | | | |
| --- | --- | --- | --- | --- |
| Variables | | Discordant relationship | Concordant relationship | P-value |
|  |  | n (%) or mean ± SD | n (%) or mean ± SD |  |
| Number of patients | | 10 | 30 |  |
| Primary diagnosus | |  |  |  |
|  | HLHS | 2 (20) | 3 (10) | 0.408 |
|  | Univentricular heart | 6 (60) | 20 (67) | 0.702 |
|  | DILV | 0 (0) | 2 (7) | 0.402 |
|  | Unbalanced AVSD | 0 (0) | 2 (7) | 0.402 |
| Associated cardiac anomaly | |  |  |  |
|  | TGA | 4 (40) | 14 (47) | 0.714 |
|  | DORV | 3 (30) | 11 (37) | 0.702 |
|  | Dextrocardia | 2 (20) | 11 (37) | 0.330 |
|  | Heterotaxy | 2 (20) | 14 (47) | 0.136 |
|  | Azygos continuation | 3 (30) | 4 (13) | 0.230 |
|  | TAPVC/PAPVC | 3 (30) | 5 (17) | 0.361 |
|  | CAVV | 4 (40) | 15 (53) | 0.465 |
|  | Dominant RV | 9 (90) | 26 (86.7) | 0.783 |
| Extracardiac anomaly | | 3 (30) | 6 (20) | 0.405 |
| Genetic anomaly | | 0 (0) | 2 (7) | 0.426 |
| Age at TCPC (year) | | 4.8±5.6 | 3.0±1.6 | 0.340 |
| Interval between BCPS and TCPC | | 3.2±3.1 | 1.7±0.6 | 0.192 |
| Weight at TCPC (kg) | | 17.6 ± 15.2 | 12.6 ± 3.6 | 0.325 |
| Height at TCPC (cm) | | 99.7±32.1 | 92.1±12.2 | 0.483 |
| Pre-TCPC ventricular dysfunction | | 1 (10) | 3 (10) | 1.000 |
| Pre-TCPC AVVR | | 2 (20) | 5 (17) | 0.810 |
| Pre-TCPC catheterization data | |  |  |  |
|  | Hemoglobin level | 17.4±4.4 | 16.6±1.7 | 0.604 |
|  | Pulmonary artery pressure (PAP) | 11.2±3.7 | 9.9±3.5 | 0.310 |
|  | Left atrial pressure (LAP) | 7.4 ± 2.7 | 6.3 ± 3.3 | 0.326 |
|  | Transpulmonary gradient (TPG) | 3.6±2.3 | 3.1±1.8 | 0.533 |
|  | Systemic ventriclar pressure (SVP) | 82.1 ± 14.0 | 82.1 ± 12.1 | 0.999 |
|  | End-diastolic pressure (EDP) | 9.3 ± 2.5 | 7.5 ± 2.5 | 0.070 |
|  | Systolic aortic pressure (SAP) | 82.9 ± 12.6 | 80.2 ± 13.0 | 0.594 |
|  | Mean aortic pressure (MAP) | 61.2 ± 14.3 | 57.8 ± 9.0 | 0.401 |
|  | Aortic oxygen saturation (SO2) | 79.3±8.0 | 86.0±5.1 | **0.007** |
|  | APC | 3 (30) | 7 (23) | 0.673 |
|  | Left PA stenosis | 4 (40) | 5 (17) | 0.126 |
| Operative data | |  |  |  |
|  | CPB time (min) | 104 ± 51 | 88 ± 43 | 0.347 |
|  | Aortic cross-clamp | 5 (50) | 12 (40) | 0.580 |
|  | Aortic cross-clamp time (min) | 38±35 | 63±29 | 0.751 |
| Postoperative data | |  |  |  |
|  | Volume administration (ml/kg) | 151 ± 146 | 159 ± 172 | 0.977 |
|  | Inotrope score | 18.5 ± 21.5 | 14.5 ± 9,8 | 0.437 |
|  | Intubation time (hours) | 52 ± 123 | 24 ± 50 | 0.494 |
|  | Re-intubation | 3 (30) | 2 (7) | 0.053 |
|  | Chest tube duration (days) | 9 ± 8 | 5 ± 3 | 0.123 |
|  | Final CVP (mmHg) | 14.0±2.7 | 13.4±2.3 | 0.489 |
| Complications | |  |  |  |
|  | Prolonged leural effusion | 5 (50) | 13 (44) | 0.714 |
|  | Chylothorax | 2 (20) | 5 (17) | 0.810 |
|  | Ascites | 3 (30) | 8 (27) | 0.838 |
|  | Secondary fenestration | 2 (20) | 0 (0) | **0.012** |
|  | Ventricular dysfunction | 2 (20) | 0 (0) | **0.012** |
| Follou-up | |  |  |  |
|  | Total deaths | 4 (40) | 1 (3) | **0.002** |
|  | In-hospital deaths | 2 (20) | 1 (3) | 0.083 |
|  | Re operation | 2 (20) | 0 (0) | **0.012** |
|  | Re-intervention | 4 (40) | 4 (13) | 0.068 |
|  | PAVM | 2 (20) | 3 (10) | 0.408 |
|  | PLE | 1 (10) | 1 (3) | 0.402 |
|  | Last SO2 | 92.0 ± 6.6 | 94.1 ± 4.8 | 0.287 |

Bold indicates P<0.05

TCPC: total cavopulmonary connection, SD: standard deviations, HLHS: hypoplastic left heart syndrome;

DILV: double-inlet left ventricle; AVSD: atrioventricular septal defect;

TGA: transposition of the great arteries; DORV: double-outlet right ventricle;

TAPVC: total anomalous pulmonary vein connection; PAPVC: partial anomalous pulmonary vein connection; CAVV: common atrioventricular valve; RV: right ventricle, BCPS: bidirectional cavopulmonary shunt,

AVVR: atrioventricular valve regurgitation, APC: aortopulmonary collaterals, PA: pulmonary artery,

CPB: cardiopulmonary bypass, CVP: central venous pressure, PAVM: pulmonary arteriovenous malformation, PLE: protein losing enteropathy, SO2: arterial oxygen saturation.

| **Table S3**: Comparison of ICU data after TCPC between patients with concordant and discordance relation | | | | |
| --- | --- | --- | --- | --- |
| Variavles | | Discordant relation | Concordant relation | P-value |
|  |  | n (%) or mean ± SD | n (%) or mean ± SD |  |
| Volume administration (ml/kg) | | 161±146 | 159±172 | 0.977 |
| Maximal inotrope score | | 18±21 | 14±10 | 0.437 |
| Ventilation time (hours) | | 52±123 | 23±49 | 0.494 |
| Chest tube drainage duration (days) | | 9±8 | 5±3 | 0.123 |
| Lactat (mg/dL) | | 4,1±2,1 | 4,0±1,2 | 0.854 |
| CRP (mg/dL) | | 72±65 | 57±41 | 0.421 |
| Leukocytes (/μl ) | | 13,4±4,3 | 15,4±5,4 | 0.326 |
| Final CVP at ICU (mmHg) | | 14,0 ±2,6 | 13,0±2,5 | 0.290 |
| MAP (mmHg) | |  |  |  |
|  | At ICU admission | 60±10 | 54±9 | 0.087 |
|  | 2 hours before extubation | 63±11 | 60±10 | 0.460 |
|  | 1 hour before extubation | 64±11 | 63±10 | 0.708 |
|  | 1 hour after extubation | 61±16 | 68±10 | 0.292 |
|  | 2 hours after extubation | 65±15 | 67±10 | 0.600 |
|  | 6 hours after extubation | 61±14 | 71±10 | **0.040** |
|  | 12 hours after extubation | 62±12 | 69±9 | 0.075 |
|  | 24 hours after extubation | 67±11 | 72±10 | 0.244 |
| Volume administration (ml/hour) | |  |  |  |
|  | At ICU admission | 217±130 | 207±162 | 0.878 |
|  | 2 hours before extubation | 131±96 | 104±99 | 0.492 |
|  | 1 hour before extubation | 88±83 | 84±83 | 0.919 |
|  | 1 hour after extubation | 81±84 | 17±31 | 0.068 |
|  | 2 hours after extubation | 109±188 | 23±48 | 0.242 |
|  | 6 hours after extubation | 54±105 | 21±48 | 0.421 |
|  | 12 hours after extubation | 25±70 | 3±10 | 0.401 |
|  | 24 hours after extubation | 69±70 | 7±24 | **0.042** |
| CO2 (mmHg) | |  |  |  |
|  | At ICU admission | 43±7 | 40±6 | 0.313 |
|  | 2 hours before extubation | 43±4 | 39±8 | **0.022** |
|  | 1 hour before extubation | 46±6 | 38±6 | **0.001** |
|  | 1 hour after extubation | 48±8 | 45±6 | 0.344 |
|  | 2 hours after extubation | 48±6 | 45±7 | 0.299 |
|  | 6 hours after extubation | 49±8 | 46±7 | 0.298 |
|  | 12 hours after extubation | 48±7 | 46±6 | 0.483 |
|  | 24 hours after extubation | 48±8 | 43±6 | 0.109 |
| CVP (mmHg) | |  |  |  |
|  | 2 hours before extubation | 18±4 | 16±4 | 0.369 |
|  | 1 hour before extubation | 18±5 | 15±3 | **0.049** |
|  | 1 hour after extubation | 18±4 | 18±5 | 0.432 |
|  | 2 hours after extubation | 16±3 | 16±4 | 0.656 |
|  | 6 hours after extubation | 17±4 | 15±4 | 0.228 |
|  | 12 hours after extubation | 14±3 | 14±3 | 0.825 |
|  | 24 hours after extubation | 14±2 | 14±3 | 0.806 |
|  | after 24 hours | 16±3 | 14±4 | 0.212 |
| Inotrope score | |  |  |  |
|  | 2 hours before extubation | 12±11 | 10±9 | 0.606 |
|  | 1 hour before extubation | 6±4 | 8±5 | 0.154 |
|  | 1 hour after extubation | 5±3 | 8±4 | 0.159 |
|  | 2 hours after extubation | 4±3 | 7±4 | 0.087 |
|  | 6 hours after extubation | 5±5 | 7±6 | 0.422 |
|  | 12 hours after extubation | 5±5 | 5±3 | 0.871 |
|  | 24 hours after extubation | 5±5 | 5±4 | 0.969 |
|  | after 24 hours | 6±7 | 4±3 | 0.543 |
| SO2 (%) | |  |  |  |
|  | At ICU admission | 93±7 | 96±4 | 0.141 |
|  | 2 hours before extubation | 91±9 | 94±4 | 0.435 |
|  | 1 hour before extubation | 90±9 | 93±4 | 0.155 |
|  | 1 hour after extubation | 90±7 | 90±6 | 0.922 |
|  | 2 hours after extubation | 91±7 | 93±4 | 0.401 |
|  | 6 hours after extubation | 92±7 | 94±4 | 0.426 |
|  | 12 hours after extubation | 92±6 | 93±5 | 0.788 |
|  | 24 hours after extubation | 92±6 | 94±5 | 0.391 |

Bold indicates P<0.05

ICU: intensive care unit, TCPC: total cavopulmonary connection, SD: standard deviations,

CRP: C-reactive protein, CVP: central venous pressure, MAP: mean arterial pressure,

CO2: partial carbon dioxide pressure, SO2: arterial oxygen saturation.
