# Supplementary material for: Flow Dynamics of Bilateral Superior Cavopulomonary Shunts Influence Outcomes After Fontan Completion
Source: Pediatr Cardiol. 2020 Mar 10;41(4):816–26. doi: 10.1007/s00246-020-02318-x (PMC7256021; doi:10.1007/s00246-020-02318-x)
Supplement: Supplementary file 13 — Supplementary file13 (DOCX 42 kb) [file 246_2020_2318_MOESM13_ESM.docx]

Figure S1: Kaplan-Meier curves comparing time to discharge from the ICU after TCPC following a unilateral or bilateral BCPS procedure. Note: the time to ICU discharge was longer in patients who underwent a bilateral BCPS procedure than in those who underwent a unilateral BCPS procedure (p=0.024, log-rank test). ICU: intensive care unit, TCPC: total cavopulmonary connection, BCPS: bidirectional cavopulmonary shunt.

Figure S2: Kaplan-Meier curves comparing estimated survival after TCPC following a unilateral or bilateral BCPS procedure. Note: survival was significantly lower in patients who underwent a bilateral BCPS procedure than those who underwent a unilateral BCPS procedure (p=0.004, log-rank test). TCPC: total cavopulmonary connection, BCPS: bidirectional cavopulmonary shunt.

Figure S3: Kaplan-Meier curves comparing estimated survival after TCPC between patients with concordant and discordant SVC/IVC configurations. Note: survival was significantly lower in patients with discordant anatomy than in those with concordant anatomy (p=0.002, log-rank test). TCPC: total cavopulmonary connection, SVC: superior vena cava, IVC: inferior vena cava.

Figure S4: Graphical presentation of the changes in MAP (A), volume administrated (B), CO2 (C), and CVP (D) in patients with discordant (red) and concordant (blue) relationship between the dominant SVC and the IVC at selected time points relative to extubation. Boxes represent the median and the lowest and highest quartiles, and whiskers indicate the minimum and maximum values. MAP: mean arterial pressure, CO2: the arterial partial carbon dioxide pressures, CVP: central venous pressure, SVC: superior vena cava, IVC: inferior vena cava.

**Video legends**

Video 1: SVC Angiogram in patients with the right dominant SVC: (A) right SVC angiogram shows right to left blood flow in the central PA; (B) left SVC angiogram shows ipsilateral blood flow into the left PA and no blood flow into the central PA. SVC: superior vena cava, PA: pulmonary artery.

Video 2: SVC Angiogram in patients with the left dominant SVC: (A) left SVC angiogram shows left to right blood flow in the central PA; (B) right SVC angiogram shows ipsilateral blood flow into the right PA and no blood flow into the central PA. SVC: superior vena cava, PA: pulmonary artery.
